# Diagnostic and prognostic value of polygene methylation detection in ascites

**DOI:** 10.3389/fgene.2025.1680036

**Published:** 2025-12-17

**Authors:** Yang Ma, Xueqing Wang, Yun Du

**Affiliations:** Department of Cytology, The Fourth Hospital of Hebei Medical University, Shijiazhuang, China

**Keywords:** DNA methylation, HOXA9, malignant ascites, RASSF1A, SEPTIN9, SHOX2

## Abstract

**Objective:**

To explore a novel combination of methylation markers for the differential diagnosis of malignant ascites (MA).

**Materials and methods:**

A cohort of 164 cancer patients and 20 patients with benign disease presenting with ascites was enrolled. Ascites was tested by means of cytopathological routine diagnosis and DNA methylation detection of SHOX2, RASSF1A, SEPTIN9 and HOXA9 in the cytological specimens. DNA methylation in bisulfite-converted DNA was determined using semi-quantitative methylation-specific real-time PCR (MS-PCR). In addition, Kaplan-Meier method was used to plot the Overall survival (OS) curve based on the methylation status of four genes to explore the relationship between gene methylation status and prognosis of patients with ascites. The Cox regression model was used to analyze the survival factors.

**Results:**

Methy-All-In-One Kit (OncoMe), a novel combination of SHOX2, RASSF1A, SEPTIN9 and HOXA9 methylation, led to an additional 29.9% increase in the detection rate of MA combined with the cytopathological test, resulting in a sensitivity of 76.2%. OncoMe showed high positive detection rates in Breast Cancer (87.5%), Pancreatic Cancer (83.3%), Gastric Cancer (79.5%), Cholangiocarcinoma (72.7%) and Ovarian Cancer (68.3%). Patients in the SHOX2 and SEPTIN9 methylation-positive groups had a significantly higher risk of disease progression than those in the negative groups. The OS of SHOX2 and SEPTIN9 gene methylation test negative group was higher than that of positive group.

**Conclusion:**

OncoMe has potential for use as a biomarker for the detection of MA. Cytological examination combined with methylation detection can greatly improve the diagnosis rate of malignant ascites. The methylation status of SHOX2 and SEPTIN9 genes is significantly correlated with the prognosis of patients with ascites.

## 1 Introduction

Malignant ascites refers to peritoneal effusion caused by primary peritoneal or other malignant tumors that metastasize to the peritoneum, often indicating that malignant tumors have reached an advanced stage and thus leading to shorter survival and lower quality of life (Li J-X. et al., 2020). Cytology is the gold standard for the diagnosis of malignant tumor cells in ascites, but its sensitivity is limited. The sensitivity of cytological detection is hindered by low tumor cell abundance and the difficult differentiation between reactive mesothelial and tumor cells. An accurate and early detection of tumor cells in the ascites fluid is of strong clinical importance in different clinical settings. The discrimination between malignant and paramalignant ascites is of importance for clinical staging and influences treatment decisions (Prat and Oncology, 2014; National Institutes of Health Consensus Development Conference Statement, 1994). A delayed etiological diagnosis will directly affect the subsequent treatment of patients and can be associated with markedly higher morbidity and mortality.

Epigenetics focuses on heritable alterations of gene expression without changing the DNA sequence of genes. DNA methylation is one of the most thoroughly studied epigenetic modifications of genomic DNA. The functions of DNA methylation modification include maintaining chromosome stability, inhibiting repetitive sequences, preventing foreign DNA integration and controlling gene expression. DNA methylation has been considered closely associated with tumorigenesis and one of the earliest molecular markers of human cancers (Li N. et al., 2020). Epigenetic changes in tumor genomic DNA are biostable and often cancer-specific alterations, which are therefore the focus of numerous ongoing research projects worldwide. DNA methylation markers have great potential for diagnosing cancer for several reasons: 1) Aberrant DNA methylation is a frequently observed characteristic of cancer cells (Jones, 2012; Baylin and Jones, 2011; Shen and Laird, 2013; Vogelstein et al., 2013; Suva et al., 2013). 2) DNA chromosomes have high chemical robustness and DNA methylation marks are stably retained during mitosis and meiosis. 3) Several analytical techniques, such as MS-PCR, allow for an accurate quantification of the respective biomarker (Dietrich et al., 2013; Dietrich et al., 2012; Jung et al., 2018).

Some studies have exhibited frequent promoter methylation in various types of tumors, including RAS association domain family 1, isoform A (RASSF1A), short stature homeobox gene two (SHOX2) (Zhang et al., 2017; Ren et al., 2017; Shi et al., 2020), homeobox A9 (HOXA9) and Septin9 (SEPTIN9) (Hulbert et al., 2017; Wang et al., 2021). HOXA9 promoter hypermethylation has been discovered in considerable proportion of tubo-ovarian high-grade serous ovarian carcinomas (Xing et al., 2015). The detection of quantitative SHOX2 and SEPTIN9 methylation levels have successfully applied to the diagnosis of colonic adenomas (Semaan et al., 2016) and the detection of malignant cells in pleural effusions (Dietrich et al., 2013) and ascites (Jung et al., 2016). Several research reported that the combination of SHOX2 and RASSF1A methylation in bronchoalveolar lavage fluid (BALF) yielded a diagnostic sensitivity of 71.5%–81.0% and a specificity of 90%–97.4% (Zhang et al., 2017; Ren et al., 2017), which led to the auxiliary diagnosis development of DNA methylation for lung cancer. According to the above preliminary research, this encourages us to apply the OncoMe to assist cytologists in the diagnosis of malignant disease, which can simultaneously detect the stations of four gene methylation, including RASSF1A, SHOX2, HOXA9 and SEPTIN9.

In our study, the detection of promotor hypermethylation of tumor suppressor genes in the ascites cellular fraction might aid greatly in the differential diagnosis of ascites. We therefore determined the promoter methylation status of the four genes in 184 patients with ascites caused by various diseases, including Gastric Cancer (GC), Ovarian Cancer (OC), Liver Cancer, Colon Cancer (CC), Pancreatic Cancer (PC), Breast Cancer (BC), Lung Cancer (LC), Renal Cancer (RC), Mesothelioma (MESO), other cancers and some benign disease, to examine whether this panel of novel methylated DNA markers could effectively identify malignant effusions. Besides, to explore the relationship between the methylation status of these genes and the prognosis of patients with ascites, in order to provide powerful auxiliary information for the accurate diagnosis of malignant ascites.

## 2 Materials and methods

### 2.1 Materials

A total of 184 ascites specimens were selected at The Fourth Hospital of Hebei Medical University from July 2021 to July 2023 for examination. Conventional cytopathological investigation and the DNA methylation of RASSF1A, SHOX2, SEPTIN9 and HOXA9 were measured in the cellular fraction of ascites specimens in this study. All enrolled patients signed informed consent for the following study. This study was conducted under the ethical principles of the Declaration of Helsinki, and was approved by the Ethics Committee of the Fourth Hospital of Hebei Medical University. Detection of malignancy was performed by biopsy or surgical specimens. Benign patients with ascites were diagnosed comprehensively by clinical and cytological methods and they did not have any evidence of cancer within the last 15 years. To minimize selection bias and enhance the representativeness of the study cohort, consecutive patients presenting with ascites during this period were considered for inclusion. The selection process was based strictly on the availability of sufficient ascites volume and complete clinical data, without prior knowledge of cytological or pathological results. The inclusion criteria for this study were: (1) The study included patients with only ascites and no other serous cavity effusions; (2) Availability of complete clinical records, including demographic data, imaging studies, and follow-up information; (3) Availability of complete clinical records, including demographic data, imaging studies, and follow-up information. The exclusion criteria included: (1) Insufficient ascites volume for both cytological and molecular analyses; (2) Incomplete clinical or follow-up data; (3) Presence of other significant serous cavity effusions that could confound the diagnosis; (4) Previous or concurrent malignancy within the last 15 years for the benign group.

### 2.2 Sampling method

Fresh ascites samples (100–200 mL) were filtered by disposable cell collector to enrich tumor cells. Then we would use the disposable cell collector to evenly smear 6–8 slides for conventional cytological smear diagnosis. Cell blocks were prepared in case of cell abundance, and immunocytochemical staining and diagnosis. After the routine diagnostics’ completion, ascites samples (10–20 mL) were fixed with 20 mL of cell prevention solution (20140074, Tellgen Co., China), and centrifuged at 4,000 rpm at 24 °C for 10 min to remove supernatant and retain residual precipitation. The pellets were dissolved in 1 mL of cell prevention solution and stored at room temperature for no more than 2 weeks.

### 2.3 DNA extraction, bisulfite treatment and methylation analysis

DNA extraction and bisulfite conversion of the cell pellets were performed using the Methy-All-In-One Kit (Tellgen Co., Shanghai, China). The concentration of extracted DNA was accurately measured using highly sensitive fluorescent dye assay (Fluo-100B, Hangzhou Allsheng Instruments Co., Ltd., China). 50 ng of DNA extracted from the sample was treated with sodium bisulfite using the Tellgen DNA Purification Kit (PF03X056, Tellgen Co., China). After purification, the bisulfite-converted DNA was amplified in parallel in two tubes by multiple methylation specific real-time PCR (MS-PCR). One MS-PCR amplifies methylated SHOX2 (VIC), RASSF1A (FAM), and ACTB (CY5) DNA, while another MS-PCR amplifies methylated HOXA9 (VIC), SEPTIN9 (FAM), and ACTB (CY5), which served as internal controls for the quantification of total input DNA.

Primer and probe sequences were as follow, the forward primer of SHOX2 was TTG​TTT​TTG​GGT​TCG​GGT​T, the reverse primer of SHOX2 was CAT​AAC​GTA​AAC​GCC​TAT​ACT​CG, the probe of SHOX2 was VIC- ATC​GAA​CAA​ACG​AAA​CGA​AAA​TTA​CC, the forward primer of RASSF1A was CGG​GGT​TCG​TTT​TGT​GGT​TTC, the reverse primer of RASSF1A was CCG​ATT​AAA​TCC​GTA​CTT​CGC, the probe of RASSF1A was FAM-TCGCGTTTGTTAGCGTTTAAAGT, the forward primer of HOXA9 was GGT​ATA​TCG​TAG​CGG​GTA​TAG​C, the reverse primer of HOXA9 was AAC​TTC​CAA​TCC​AAA​ACG​ACG, the probe of HOXA9 was VIC-CCCTCCTAACCAACTCCTCCGTAA, the forward primer of SEPTIN9 was GTT​TTG​TAT​TGT​AGG​AGC​GC, the reverse primer of SEPTIN9 was CGA​AAA​AAC​GCC​CCC​GAC​GA, the probe of SEPTIN9 was FAM-AACCCTACGCGCTAA.

The positive quality controls were plasmids containing the methylated DNA of SHOX2, RASSF1A, SEPTIN9 and HOXA9 that have no bioactivity. PCR amplification was performed in an ABI 7500 Real-Time PCR instrument (Applied Biosystems, CA, USA), and SDS Software (Applied Biosystems) was used to obtain the results of the analysis. Co-methylation levels of a gene of interest were expressed by △Ct, where △Ct = Ct (gene of interest) - Ct (internal control). Samples were included in the analysis when 18 ≤ Ct_-ACTB_ ≤30. Samples were classified as methylation positive when at least one of the four genes’ DNA methylation levels correspondingly met the following quantitative criteria: Ct_SHOX2_< 32 and △Ct_SHOX2_ ≤ 9; Ct_RASSF1A_< 35 and △Ct_RASSF1A_ ≤ 12; Ct_SEPTIN9_ < 35 and △Ct_SEPTIN9_ ≤ 9; and Ct_HOXA9_ < 32 and △Ct t_HOXA9_ ≤ 8. All others were classified as methylation negative. The cutoff criteria for methylation positivity were established based on pre-experimental validation and prior studies using the same methylation panel, aiming to maximize specificity while maintaining diagnostic sensitivity.

### 2.4 Statistical analysis

Statistical analyses were performed using the SPSS 26.0 software package (SPSS Inc., Chicago, IL). The frequency of methylation in the SHOX2, RASSF1A, SEPTIN9 and HOXA9 genes was analyzed using the Chi-square test and Fisher exact probability method. To evaluate the efficacy of gene methylation in ascites diagnosis, we constructed a Receiver Operating Characteristic curve (ROC) and calculated the Area Under the Curve (AUC). We used the Cox proportional hazards model (Cox model) to explore the influencing factors and included variables with a P value less than 0.05 in the univariate Cox regression analysis into the multifactor analysis to adjust for potential confounders. In addition, Kaplan-Meier survival analysis was used to plot the overall survival curve.

## 3 Results

### 3.1 Methylation frequency and association with clinicopathologic features in ascites samples

Patients’ characteristics are summarized in Table 1. 164 cases were diagnosed as malignant tumors, including 44 GC, 41 OC 18 CC, 12 PC, 11 CGC, 9 liver cancer, 8 BC, 8 lung cancer, 3 MESO and three other malignant carcinoma cases. The other 20 benign disease cases include 10 liver cirrhosis, 5 nephropathy, 3 tuberculosis and two other benign disease cases. There was no significant difference in gender and age between malignant ascites and benign ascites (*P* > 0.05, Table 2).

**TABLE 1:** Baseline characteristics of patients.

Classification	Tumor classification	n	Age (years)		Gender	
			Range	Mean ± SEM	Male (%)	Female (%)
MA	GC	44	33–82	58.9 ± 11.2	33 (75.0)	11 (25.0)
	OC	41	38–86	63.8 ± 10.3	0 (0)	41 (100.0)
	CC	18	33–83	60.7 ± 12.8	11 (61.1)	7 (38.9)
	PC	12	47–85	62.7 ± 11.8	8 (66.7)	4 (33.3)
	CGC	11	46–88	62.9 ± 12.5	8 (72.7)	3 (27.3)
	LC#	9	35–71	54.9 ± 11.2	9 (100)	0 (0)
	BC	8	42–67	57.8 ± 8.0	0 (0)	8 (100)
	LC*	8	38–76	58.6 ± 13.8	5 (62.5)	3 (37.5)
	MESO	3	49–68	57.7 ± 9.7	1 (33.0)	2 (66.7)
	Other MA	0	34–80	64.0 ± 13.5	7 (70.0)	3 (30.0)
BA	LCH	10	41–85	70.5 ± 13.0	7 (63.6)	3 (36.4)
	NP	5	55–69	61.8 ± 6.5	8 (80.0)	1 (20,0)
	TC	3	39–72	59.3 ± 17.8	1 (33.3)	2 (66.7)
	Other BA	2	41–50	45.5 ± 6.4	1 (50.0)	1 (50,0)

MA, Malignant ascites; BA, Benign ascites; GC, Gastric Cancer; OC, Ovarian Cancer; CC, Colorectal Cancer; PC, Pancreatic Cancer; CGC, Cholangiocarcinoma; LC#, Liver Cancer; BC, Breast Cancer; LC*, Lung Cancer; MESO, Mesothelioma; LCH, Liver cirrhosis; NP, Nephropathy; TC, Tuberculosis.

**TABLE 2:** Comparison of general characteristics between malignant and benign ascites.

Characteristics		MA	BA	$x^{2}$	P
Gender	Male	82 (50.0%)	13 (65.0%)	1.606	0.205
	Female	82 (50.0%)	7 (35.0%)		
Age	≤60	79 (48.2%)	7 (35.0%)	1.242	0.265
	>60	85 (51.8%)	13 (65.0%)		

*P < 0.05, The difference was statistically significant.

**FIGURE 1:**
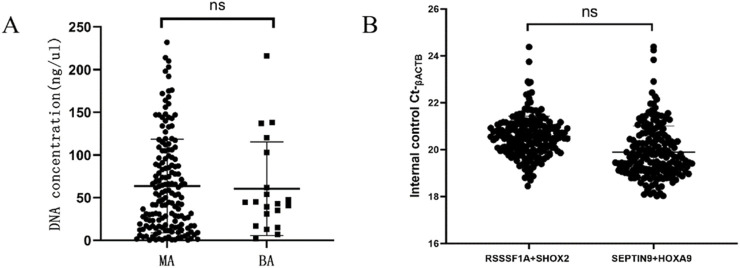
The DNA concentration of ascites samples. **(A)** The distribution of DNA concentration of ascites samples. **(B)** The distribution of Ct values of β-actin (ACTB) gene. ns, no significance; Ct, cycle threshold.

To confirm the cancer specificity of the promoter methylation events of these four genes, a quantitative methylation-specific real-time PCR (MS-qPCR) assay for each of the genes was conducted on the ascites samples of 164 malignant tumor patients and 20 benign disease patients. The DNA concentrations of these ascites samples ranged from 0.32 ng/μL to 232 ng/μL. The DNA concentration of each sample was determined and amplified at a concentration of 10 ng/μL unless the total amount of DNA was insufficient. As an internal control, the Ct value of ACTB fluctuated from 18.02 to 24.39. Thus, valid measurements were successfully obtained for all ascites samples (Figure 1).

**FIGURE 2:**
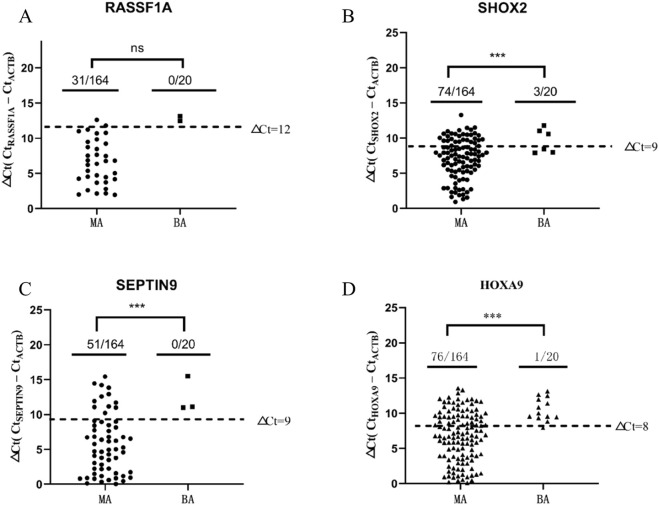
Comparison of Gene Methylation Positive Rates between Malignant and Benign Ascites. **(A)** The methylation level of RASSF1A in the tested specimens. **(B)** The methylation level of SHOX2 in the tested specimens. **(C)** The methylation level of SEPTIN9 in the tested specimens. **(D)** The methylation level of HOXA9 in the tested specimens. △Ct, delta cycle threshold. ***P < 0.05.

By using the optimal methylation cutoff value for individual genes, the positive detection rates of each marker in malignant tumor patients ranked from high to low were 46.3% (HOXA9), 45.1% (SHOX2), 31.1% (SEPTIN9) and 18.9% (RASSF1A). Combined with four genes, the positive detection rates were greatly improved to 70.1%, which can significantly improve the diagnosis rate. Meanwhile in benign disease samples, the positive detection rates of SHOX2 and HOXA9 were 15.0% and 5.0% respectively, while that of RASSF1A and SEPTIN9 were both 0%. The positive detection rate of the four genes combination was 20.0%. The positive rate of individual methylation (SHOX2, SEPTIN9, HOXA9) and the four combined methylation genes were significantly different between benign and malignant cases. Whereas the significance of positive methylation of RASSF1A was unclear (Table 3; Figure 2).

**TABLE 3:** The methylation results of genes in malignant and benign ascites.

Classification	n	RASSF1A (%)	SHOX2 (%)	SEPTIN9 (%)	HOXA9 (%)	OncoMe (%)
MA	164	31 (18.9)	74 (45.1)	51 (31.1)	76 (46.3)	115 (70.1)
BA	20	0 (0)	3 (15.0)	0 (0)	1 (5.0)	4 (20.0)
$x^{2}$	​	3.297	6.646	8.604	12.520	19.601
P	​	0.069	0.010*	0.003*	0.000*	0.000*

*P < 0.05, The difference was statistically significant.

For all malignant tumor patients’ samples, the clinical performance of the methylation events of these four genes with regard to pathologically determined histological classification was analyzed and was detailed in Table 4. Gastric and ovarian cancer were the most common cancer types in our study, and the positive rates of four-gene methylation combined detection were 79.5% and 68.3% respectively. The positive rate of four-gene methylation combined detection in colorectal cancer patients was 61.1%. Furthermore, the positive combined detection rates in other types of carcinomas ranked from high to low were BC (87.5%), PC (83.3%), CC (72.7%), LC (50.0%) and MESO (33.3%) respectively.

**TABLE 4:** The expression of cytology and gene methylation in different cancers.

Tumor classification	n	Cytology (%)	RASSF1A (%)	SHOX2 (%)	SEPTIN9 (%)	HOXA9 (%)	OncoMe (%)
GC	44	24 (54.5)	5 (11.4)	23 (52.3)	23 (52.3)	26 (59.1)	35 (79.5)
OC	41	28 (68.3)	8 (19.5)	17 (41.5)	3 (7.3)	21 (51.2)	28 (68.3)
CC	18	3 (16.7)	1 (5.6)	4 (22.0)	8 (44.4)	2 (11.1)	11 (61.1)
PC	12	5 (41.1)	4 (33.3)	5 (41.7)	4 (33.3)	6 (50.0)	10 (83.3)
CGC	11	5 (45.5)	1 (9.1)	6 (54.4)	3 (27.3)	6 (54.5)	8 (72.7)
LC#	9	1 (11.1)	1 (11.1)	1 (11.1)	1 (11.1)	1 (11.1)	2 (22.2)
BC	8	4 (50.0)	7 (87.5)	6 (75.0)	6 (75.0)	5 (62.5)	7 (87.5)
LC*	8	2 (25.0)	1 (12.5)	3 (37.5)	1 (12.5)	4 (50.5)	4 (50.0)
MESO	3	2 (66.7)	0 (0)	1 (33.3)	1 (33.3)	1 (33.3)	1 (33.3)
Others	10	2 (20.0)	3 (30.0)	8 (80.0)	1 (10.0)	4 (40.0)	9 (90.0)
Total	164	76 (46.3)	31 (18.9)	74 (45.1)	51 (31.1)	76 (46.3)	115 (70.1)

GC, Gastric Cancer; OC, Ovarian Cancer; CC, Colorectal Cancer; PC, Pancreatic Cancer; CGC, Cholangiocarcinoma; LC#, Liver Cancer; BC, Breast Cancer; LC*, Lung Cancer; MESO, Mesothelioma.

### 3.2 Comparison of the diagnostic value of gene methylation and cytology in ascites

**FIGURE 3:**
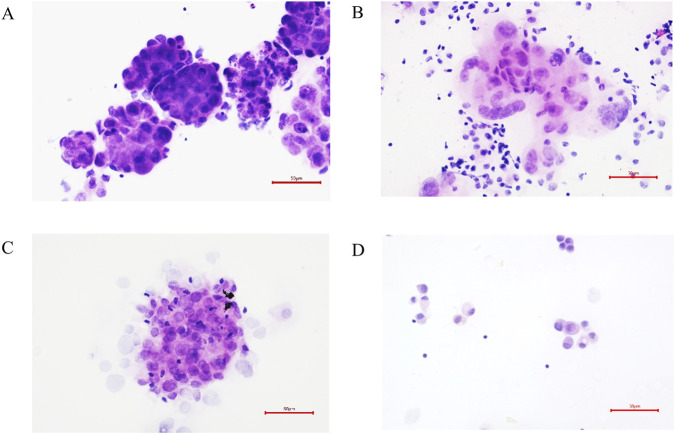
Examples of cytological diagnosis results. **(A,B)** Cancer cells (400×); **(C)** Atypical cells (400×). **(D)** Mesothelial cells (400×).

All specimens were routinely and immunohistochemically diagnosed by two cytopathologists. Among the 164 malignant tumor patients’ samples, 49 contained tumor cells, 27 contained suspected tumor cells, and 88 did not contain tumor cells. Meanwhile none of the 20 benign samples contained tumor cells (Figure 3). Table 5 shows the positive rates of four-gene methylation in different cytological diagnostic groups. Of the 164 MA samples, 49 were cytologically definitively diagnosed as malignant, of which 42 were positive (85.7%) for the gene combination test. Of the 27 suspected positive samples, 24 were positive (88.9%). Of the 88 cytologically negative samples, 49 were positive (54.4%). Therefore, the gene combination test significantly improved the diagnostic accuracy of the cytological suspect positive and negative group.

**TABLE 5:** Gene methylation in different cytology diagnostic groups.

Cytological detection	RASSF1A	SHOX2	SEPTIN9	HOXA9	OncoMe
MA (n = 164)	31	74	51	76	115
Positive (49)	11 (22.4%)	29 (59.2%)	20 (40.8%)	36 (73.5%)	42 (85.7%)
Suspicious (27)	6 (22.2%)	14 (51.9%)	16 (59.3%)	18 (66.7%)	24 (88.9%)
Negative (88)	14 (15.9%)	31 (35.2%)	15 (17.0%)	22 (25.0%)	49 (54.5%)
BA (20)	0	3	0	1	4
Positive (0)	0	0	0	0	0
Negative (20)	0	3 (15.0%)	0	1 (5.0%)	4 (20.0%)

In this study, ROC curves were plotted to evaluate the diagnostic performance of RASSF1A, SHOX2, SEPTIN9 and HOXA9 gene methylation detection and cytopathological detection in differentiating benign and malignant ascites. The sensitivity and specificity of conventional ascites cytopathological detection were 46.3% and 100%, respectively. The sensitivities of RASSF1A, SHOX2 and SEPTIN9 methylation tests were 18.9%, 45.1% and 31.1%, respectively, which were lower than those of cytopathological tests. The sensitivity of HOXA9 methylation detection was 46.3%, which was comparable to that of cytopathological detection. The sensitivity of four-gene methylation combined detection reached 70.1%, which was significantly higher than that of cytopathological detection. When the cytopathological test was combined with the four-gene methylation test, the sensitivity was further improved to 76.2%, which was the best performance of all the tests.

**FIGURE 4:**
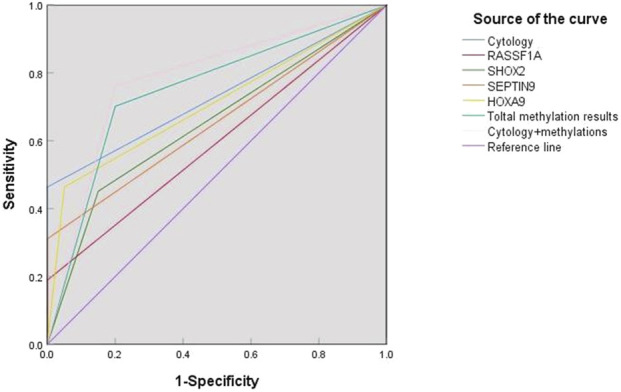
Comparison of diagnostic values of various methods for ascites detection.

The AUC values of cytopathology, individual gene methylation detection, four gene combination detection, and cytopathology and four gene combination detection were 0.732, 0.595, 0.652, 0.655, 0.707, 0.751 and 0.781 respectively. As shown in Table 6, the sensitivity of ascites diagnosis could be increased to 76.2% and the AUC value also increased to 0.781 by combining conventional cytopathology with gene methylation detection. Therefore, it can be concluded that four gene methylation detection in ascites can be used as an effective auxiliary mean for cytopathological diagnosis, thereby improving diagnostic sensitivity (Table 6; Figure 4).

**TABLE 6:** Comparison of the diagnostic efficacy of gene methylationand cytology in ascites diagnosis.

Diagnostic method	AUC		Sensitivity (95% CI)	Specificity (95% CI)	Positive predictive value	Negative predictive value	P
	Value	95% CI					
Cytology	0.732	0.647–0.816	46.3% (38.4%–54.4%)	100% (83.2%–100%)	100%	18.5%	0.001*
RASSF1A	0.595	0.480–0.709	18.9% (13.2%–26.0%)	100% (83.2%–100%)	100%	13.1%	0.168
SHOX2	0.652	0.536–0.765	45.1% (37.3%–53.2%)	85.0% (62.1%–96.8%)	96.1%	15.8%	0.028*
SEPTIN9	0.655	0.554–0.757	31.1% (24.1%–38.9%)	100% (83.2%–100%)	100%	15.0%	0.023*
HOXA9	0.707	0.610–0.803	46.3% (38.4%–54.4%)	95.0% (75.1%–99.9%)	98.7%	17.8%	0.003*
OncoMe	0.751	0.640–0.861	70.1% (62.4%–77.1%)	80.0% (56.3%–94.3%)	96.6%	24.6%	0.000*
Cytology+ OncoMe	0.781	0.673–0.890	76.2% (68.9%–82.5%)	80.0% (56.3%–94.3%)	96.9%	29.1%	0.000*

*P < 0.05, The difference was statistically significant.

### 3.3 Relationship between the methylation of RASSF1A, SHOX2, SEPTIN9, HOXA9 and the prognosis of patients with malignant ascites

We performed the univariate Cox regression analysis for prognostic effects of ascites. It was identified male gender (HR = 0.684, *P* = 0.038), SHOX2 methylation (HR = 1.699, *P* = 0.003), SEPTIN9 methylation (HR = 1.688, *P* = 0.006), and OncoMe positivity (HR = 1.492, *P* = 0.046) as significant predictors of survival. In multivariate analysis, male gender (HR = 0.593, *P* = 0.005), SHOX2 methylation (HR = 1.806, *P* = 0.013), and SEPTIN9 methylation (HR = 1.710, *P* = 0.011) remained independent prognostic factors, while OncoMe lost significance (*P* = 0.518). RASSF1A, HOXA9, and age showed no significant association with survival. That is to say, there were significant differences in overall survival (OS) between methylation-positive and negative patients among four-gene methylation combined group, the individual SHOX2 group and the individual SEPTIN9 group. It was suggested that SHOX2, SEPTIN9 and four-gene methylation combined detection were important factors affecting the prognosis of patients with ascites. In addition, the variables of SHXO2, SEPTIN9 and four-gene combined detection were included to construct Cox proportional risk model. The analysis indicated that methylation of SHOX2 and SEPTIN9 were independent factors affecting the prognosis of patients with ascites (Table 7). In the multivariate Cox regression analysis, we adjusted for all available clinical variables that were significantly associated with survival in the univariate analysis, including gender, SHOX2 methylation, SEPTIN9 methylation, and OncoMe positivity. However, due to the retrospective nature of this study and limitations in data availability, we were unable to incorporate tumor stage or treatment history into the model. Future prospective studies with more comprehensive clinical data are warranted to validate these findings.

**TABLE 7:** Cox regression analysis of prognostic factors in patients with ascites.

Characteristics	n	Unifactor cox analysis			Multivariate cox analysis		
		HR	95% CI	P	HR	95% CI	P
Gender							
Male	95	0.684	0.478–0.980	0.038*	0.593	0.411–0.855	0.005*
Female	89						
Age (years)							
≤60	86	1.162	0.815–1.659	0.407	​	​	​
>60	98				​	​	​
RASSF1A							
+	31	0.890	0.551–1.437	0.633	​	​	​
−	153				​	​	​
SHOX2							
+	77	1.699	1.191–2.424	0.003*	1.806	1.131–2.884	0.013*
−	107						
SEPTIN9							
+	51	1.688	1.163–2.448	0.006*	1.710	1.131–2.588	0.011*
−	133						
HOAX9							
+	77	1.398	0.981–1.993	0.064	​	​	​
−	107				​	​	​
OncoMe							
+	119	1.492	1.007–2.211	0.046*	0.831	0.475–1.455	0.518
−	65						

*P < 0.05, The difference was statistically significant.

**FIGURE 5:**
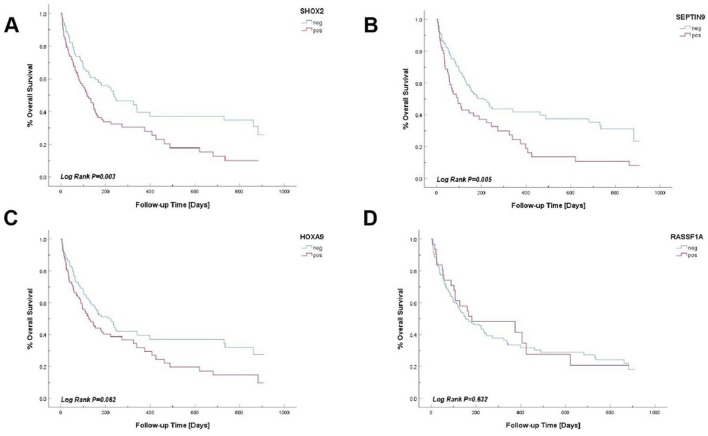
OS curves for gene methylation. **(A)** OS Curve for SHOX2 Gene Methylation. **(B)** OS Curve for SEPTIN Gene Methylation. **(C)** OS Curve for HOXA9 Gene Methylation. **(D)** OS Curve for RASSF1A Gene Methylation.

Kaplan-Meier method was used to plot the total survival curves of RASSF1A, SHOX2, SEPTIN9 and HOXA9 methylation states. The overall survival of SHOX2 and SEPTIN9 gene methylation test negative group was higher than that of positive group (P < 0.05). However, there was no statistical significance between the methylation status of RASSF1A and HOXA9 genes and overall survival (P > 0.05, Figure 5).

## 4 Discussion

Despite its role as the preferred method, cytology’s diagnostic sensitivity for distinguishing benign from malignant ascites is severely limited when tumor cells are scarce or morphologically unremarkable, often necessitating repeated sampling without achieving a definitive diagnosis (Rahemi et al., 2021; Liu et al., 2014). In contrast, DNA methylation biomarkers such as RASSF1A and SHOX2, detectable in various body fluids and linked to early tumorigenesis, have emerged as promising complementary tools for this differentiation (Raj and Kumar, 2023; Zhang et al., 2023).

In this study, we effectively used polygene methylation detection to assist the diagnosis of ascites. Compared with traditional abdominal biopsy, this detection method has advantages of simple operation, short reaction time and lightening patients’ burden. In this experiment, the DNA concentration of ascites was sufficient in most cases, the concentration distribution range was wide, ranging from 1.67 ng/μL to 232 ng/μL, and the difference between the DNA concentration of MA and BA was not statistically significant. Through DNA concentration determination, the measured value of the internal control gene ACTB in this study has a very small fluctuation range. Valid measured values were obtained in 164 MA and 20 BA samples (18.02 ≤ Ct-ACTB ≤ 24.39), which was consistent with the results of Liang et al. (2022), indicating that DNA could be effectively extracted from the ascites samples and successfully tested for subsequent methylation. However, no significant correlation was found between DNA concentration and ascites type. Furthermore, combining previous research (Liang et al., 2022) and repeated pre-experiments, the cutoff criterion of the OncoMe methylation assay for the diagnosis of ascites was established, and these criteria provide high specificity at the expense of sensitivity (Table 6). Specimens were classified as methylation positive when at least one of the four genes’ DNA methylation values met the below quantitative criteria: Ct_SHOX2_ < 32 and △Ct _SHOX2_ ≤ 9; Ct_RASSF1A_ < 35 and △Ct _RASSF1A_ ≤ 12; Ct_SEPTIN9_ < 35 and △Ct _SEPTIN9_ ≤ 9; and Ct_HOXA9_ < 32 and △Ct _HOXA9_ ≤ 8. All others were classified as methylation negative. Meanwhile, the complexity of ascites samples also brings certain challenges to the detection. How to further optimize the sample processing and analysis process to improve the sensitivity and specificity of the detection need more research and exploration.

Our findings with the OncoMe panel (SHOX2, RASSF1A, SEPTIN9, and HOXA9) in malignant ascites are consistent with and extend previous reports on DNA methylation biomarkers in body fluids. For instance, Jung et al. evaluated SHOX2 and SEPTIN9 methylation in ascites and reported that the combination of both markers increased the detection rate of malignant ascites by 42% when combined with cytology, achieving a sensitivity of 37% at 97% specificity in a cohort of 134 cancer patients (Jung et al., 2016). In this study, the detection rate of cytology was 46.3%, and the methylation detection rates of RASSF1A, SHOX2, SEPTIN9 and HOXA9 genes in malignant ascites samples were 18.9%, 45.1%, 31.1% and 46.3% respectively. However, the four-gene OncoMe panel alone reached a sensitivity of 70.1%, and when combined with cytology, further improved sensitivity to 76.2%, with a specificity of 80%. This suggests that the inclusion of RASSF1A and HOXA9, in addition to SHOX2 and SEPTIN9, may enhance diagnostic sensitivity, particularly in cytologically challenging cases. Moreover, the prognostic value of SHOX2 and SEPTIN9 methylation observed in our study aligns with Jung et al., who also identified these markers as independent prognostic factors in ascites (Jung et al., 2016). Thus, the OncoMe panel not only maintains the prognostic strengths of prior biomarker sets but also offers improved diagnostic performance, supporting its potential as a ancillary tool in the clinical evaluation of ascites. Beyond ascites, studies in pleural effusions have also highlighted the utility of methylation biomarkers. Dietrich et al. reported that SHOX2 and SEPTIN9 methylation in pleural effusions provided a diagnostic sensitivity of 26% and 100% specificity, with combined cytology and methylation analysis increasing detection by 71% (Dietrich et al., 2013). More recently, Liang et al. applied a similar four-gene panel (OncoMe) to pleural effusions and achieved a sensitivity of 85% and specificity of 94%, underscoring the cross-cancer applicability of these markers (Liang et al., 2022). The positive rates of individual methylation (SHOX2, SEPTIN9, HOXA9) and the four combined methylation genes were significantly different between benign and malignant cases. Our study builds on this foundation by validating the OncoMe panel specifically in ascites, a fluid with distinct biological and cellular characteristics. The improved diagnostic performance observed in our cohort particularly in common MA etiologies such as gastric, ovarian, and pancreatic cancers support the panel’s utility as a complementary tool to cytology. Meanwhile the positive rate of RASSF1A was too low and relatively no diagnostic value in this study. But the methylation positive rate of RASSF1A was statistically significant in the studies of pleural fluid (Zhang et al., 2023) and bronchial lavage fluid (Zhang et al., 2017). The reason for this difference may be related to tissue specificity, and different types of body fluids may have different biological characteristics, including cell composition, immune environment, microbial communities, which may affect the pattern of DNA methylation and its sensitivity in disease diagnosis.

In summary, while previous studies established the diagnostic and prognostic value of SHOX2 and SEPTIN9 in both ascites and pleural effusions, our study introduces the novel combination of four genes (SHOX2, RASSF1A, SEPTIN9, and HOXA9) in ascites, demonstrating superior diagnostic sensitivity without compromising specificity. This multi-marker approach not only improves detection rates but also reinforces the prognostic stratification of patients with MA, offering a clinically viable adjunct to standard cytological evaluation.

Gastric cancer and ovarian cancer are the most common cause of MA, accounting for approximately 1/2 of MA cases, followed by Colon cancer, Pancreatic cancer and cholangiocarcinoma, etc, which is consistent with Zha et al. (2024). It was found in this study that there was no statistical significance in gender and age, which was consistent with the results of Jung et al. (2016). Compared to cytological diagnosis, the combined detection rate of four-gene methylation in Gastric Cancer and Colorectal Cancer were further improved from 54.5% to 79.5% and 16.7%–61.1%, in addition, that of Pancreatic cancer, cholangiocarcinoma, Breast cancer, Liver cancer and Lung Cancer all had a certain improvement. Strangely, OncoMe showed a very low detection sensitivity (33.3%) in MA caused by mesothelioma (MESO). On account of the incidence rate of MESO is low in China, thus we’ve only had three cases in the study. Malignant mesothelioma (MM) is an aggressive cancer and frequently appear with recurrent hemorrhagic or inflammatory effusions, which mask the incipient stages of the disease and thereby delay the diagnosis. We need to increase the number of specimens to explore further reliable diagnostic biomarkers (Bibby et al., 2016).

In addition, we explored the potential value of DNA methylation in assessing the prognosis of patients with ascites. The results showed that patients with positive methylation of SHOX2 and SEPTIN9 genes had a significantly higher risk of disease progression than patients with negative methylation, and their overall survival was significantly shortened. DNA methylation of SHOX2 and SEPTIN9 can be used as an adjunct tool in cytological diagnosis and is expected to play an important role in prognostic assessment. This finding is consistent with the results of the existing literature (Chen et al., 2022; Dietrich et al., 2023; Fleischhacker et al., 2023; Faaborg et al., 2022), that is, DNA methylation is associated with the presence of malignant tumors and poor prognosis. However, the association between the methylation status of RASSF1A and HOXA9 genes and patient prognosis was not significant, suggesting that the correlation between the methylation status of different genes and patient prognosis may be different. In this study, when different types of cancer were statistically analyzed together for the prognosis, there were indeed some complexities and potential biases. Although different types of cancer have different biological characteristics and clinical behaviors, there are still common molecular characteristics in different cancers, and the analysis of cross-cancer species is valuable. In particularly, this approach is reasonable when looking for universal biomarkers across multiple cancer types, which was confirmed in the study of Jung et al. (Jung et al., 2016).

This study evaluated the diagnostic performance of these gene methylations in ascites by mapping ROC curves. It was found that the AUC values of RASSF1A, SHOX2, SEPTIN9 and HOXA9 gene methylation and four-gene combined detection and cytology combined detection were 0.595, 0.652, 0.655, 0.707, 0.751 and 0.781, respectively. These results indicate that the gene methylation test has a high diagnostic efficacy in distinguishing MA from BA, and the combined test has the best diagnostic efficacy. The sensitivity and specificity of the combined cytology and four-gene test for the diagnosis of ascites increased to 76.2% and 80%. Our data indeed demonstrated that SHOX2 and SEPTIN9 methylation statuses were independent prognostic factors for patients with malignant ascites (MA) in both univariate and multivariate Cox analyses (*P* < 0.05, Table 7). In contrast, RASSF1A and HOXA9 methylation did not show significant associations with OS (*P* > 0.05). Kaplan-Meier survival curves further validated these findings (Figure 5). A simplified model incorporating only SHOX2 and SEPTIN9 could enhance clinical utility by reducing complexity while retaining prognostic accuracy. However, the original four-gene panel (OncoMe) was designed primarily for diagnostic sensitivity, where combining multiple markers (including HOXA9 and RASSF1A) improved detection rates. In summary, compared with conventional detection methods, combined detection shows better diagnostic performance. While SHOX2 and SEPTIN9 are sufficient for prognostic stratification, the full OncoMe panel remains critical for maximizing diagnostic sensitivity.

There are still some deficiencies which need to be further perfected: First, the retrospective design restricted our ability to collect comprehensive clinical variables such as tumor stage, treatment history, and comorbidities, which may influence both methylation status and patient survival. Second, the single-center nature of the study may introduce selection bias and limit the generalizability of our findings. Third, the small sample size of the benign ascites group (n = 20) compared to the malignant group (n = 164) may affect the reliability of specificity estimates and the overall diagnostic performance of the methylation panel. Fourth, the heterogeneity of cancer types included in the study, while reflective of real-world clinical scenarios, complicates the interpretation of methylation patterns and prognostic analyses across different malignancies. In particular, rare cancers such as mesothelioma were underrepresented, which may have led to underestimation of the test’s diagnostic sensitivity in these subtypes. Finally, the methylation cutoff values used in this study, though based on pre-experimental validation and prior literature, were derived from the same dataset and may be subject to overfitting. External validation in an independent, multi-center cohort is necessary to confirm the robustness and general applicability of these thresholds. Future prospective studies with larger cohorts will incorporate these parameters to further validate our findings and explore their interactions with methylation biomarkers. We also plan to address these important questions in future multi-center studies with larger and more balanced cohorts, which will allow for robust stratified survival analyses.

In conclusion, we found that the aberrant promotor methylation of OncoMe (SHOX2, RASSF1A, SEPTIN9 and HOXA9) was a cancer-specific alteration and might be a valuable marker to aid in the differentiation of MA. OncoMe routine testing could help clinicians quickly identify MA and facilitate clinical decision-making.

## Data Availability

The original contributions presented in the study are included in the article/supplementary material, further inquiries can be directed to the corresponding author.
